# A Rare Case of a Large Intra-Abdominal Lymphatic-Venous Malformation in a Young Woman Presenting to the Emergency Room with Abdominal Pain

**DOI:** 10.3390/reports8030183

**Published:** 2025-09-18

**Authors:** Gloria Perillo, Domiziana Santucci, Raffaele Ragone, Elva Vergantino, Stefania Lamja, Linda Celozzi, Bruno Beomonte Zobel, Eliodoro Faiella

**Affiliations:** Research Unit of Radiology and Interventional Radiology, Department of Medicine and Surgery, Università Campus Bio-Medico di Roma, Via Alvaro del Portillo 21, 00128 Roma, Italy; d.santucci@policlinicocampus.it (D.S.); elva.vergantino@unicampus.it (E.V.); s.lamja@policlinicocampus.it (S.L.); l.celozzi@unicampus.it (L.C.); b.zobel@policlinicocampus.it (B.B.Z.); e.faiella@policlinicocampus.it (E.F.)

**Keywords:** lymphatic-venous malformation, abdominal mass, CT scan, MRI, ISSVA 2018, biopsy, histology, surgery

## Abstract

**Background and Clinical Significance**: Lymphatic-venous malformations (LVMs) are uncommon congenital vascular anomalies with low blood flow, consisting of atypical connections between lymphatic and venous vessels. They may develop in different body regions, with a predilection for lymphatic-rich areas. Fewer than 5% of LVMs are located intra-abdominally, typically arising from the mesentery, retroperitoneum, or greater omentum. Patients with intra-abdominal LVMs may be asymptomatic, but they can also present with symptoms such as acute abdominal pain, chronic discomfort, palpable masses, or progressive abdominal distension. **Case Presentation**: This case describes a 24-year-old female who presented to our emergency department with progressive abdominal distension, nausea, and vomiting. **Conclusions**: The diagnosis of LVMs can be challenging and requires a combination of imaging techniques, including ultrasound (US), computed tomography (CT), and magnetic resonance imaging (MRI), along with histological confirmation.

## 1. Introduction and Clinical Significance

Lymphatic-venous malformations (LVMs) are rare congenital low-flow vascular anomalies characterized by abnormal, interconnected lymphatic and venous vessels [1].

These malformations can occur anywhere in the body, but they are most commonly found in areas rich in lymphatic tissue, such as the head and neck, axilla, mediastinum, and groin [1]. Fewer than 5% of lymphatic malformations (LMs) are located intra-abdominally, typically arising from the mesentery, retroperitoneum, or greater omentum [2].

However, the incidence of intra-abdominal LVMs has not been documented in the literature.

Patients with intra-abdominal LVMs may be asymptomatic, but they can also present with symptoms such as acute abdominal pain, chronic discomfort, palpable masses, or progressive abdominal distension [3]. The diagnosis of LVMs can be challenging and requires a combination of imaging techniques, including ultrasound (US), computed tomography (CT), and magnetic resonance imaging (MRI), along with histological confirmation.

This case describes a 24-year-old female who presented to our Emergency Department with progressive abdominal distension, nausea, and vomiting.

Imaging revealed a giant intra-abdominal mass with compressive-displacement effects, which was subsequently diagnosed as a combined lymphatic-venous malformation based on histological and immunohistochemical findings, in accordance with the 2018 ISSVA classification [4].

## 2. Case Presentation

A 24-year-old woman presented to the Emergency Department of our hospital in January with a history of abdominal distension that had begun the previous summer. Her symptoms had progressively worsened since November, with increasing abdominal distension. On the day of presentation, she reported a general feeling of malaise accompanied by vomiting.

On physical examination, the patient was afebrile and hemodynamically stable. Her abdomen was tense but palpably soft, with dullness to percussion noted in the left upper quadrant.

Laboratory investigations revealed only moderate anemia with a hemoglobin level of 9.5 g/dL.

**Figure 1:**
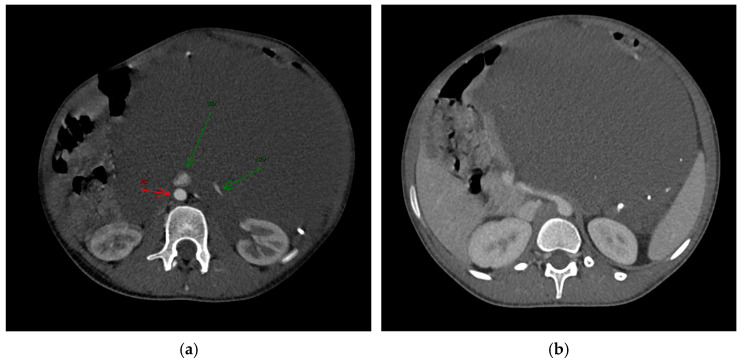
The axial post-contrast CT in the arterious (**a**) and portovenous (**b**) phase reveals the full extent of the mass and its intra-peritoneal location, without infiltration of the adjacent structures. The mass contains calcified density components, which were later identified as phleboliths. (**a**): Green arrows indicated the Renal Vein (RV) and Inferior Vena Cava (ICV); red arrow indicated the Aorta (AO).

An ultrasound examination was performed with the visualization of a multiloculated lesion, characterized by anechoic and hypoechoic cystic spaces separated by thin septations, with moderate intralesional vascularization observed after color Doppler evaluation. Then, a second-level imaging with contrast-enhanced CT scan of the abdomen was performed, revealing an intraperitoneal large, multiseptated hypodense mass containing multiple calcified components (Figure 1), measuring approximately 21 × 16 cm in the axial plane and extending up to 30 cm in the sagittal plane. The lesion occupied nearly the entire abdominal cavity, predominantly on the left side, compressing adjacent structures. It wrapped smoothly around the left kidney and adrenal gland, displacing bowel loops and other abdominal organs, with a poorly defined interface with the surrounding tissues.

The major abdominal vessels, including the celiac trunk, superior mesenteric artery, inferior mesenteric artery, and the inferior vena cava, as well as both renal veins and arteries, were compressed with a partial course within the mass itself. However, no evidence of vascular compromise was observed.

**Figure 2:**
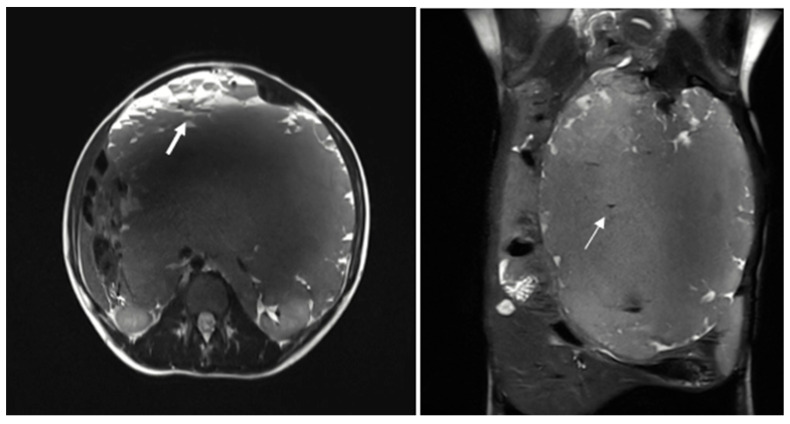
The multiloculated mass shows variable signal intensity on T2 sequences and presents cystic formations peripherally, exhibiting fluid-debris levels (thick arrow). Some calcified spots with low T2WI signals were also noted within the mass (thin arrow).

The patient was subsequently admitted for further evaluation, and an abdominal MRI was performed. Imaging sequences included T2-weighted sequences and T1-weighted sequences, with and without fat suppression. However, due to limited patient compliance, the study was interrupted before the contrast agent administration. Despite these limitations, MRI confirmed the presence of a large multiloculated mass with peripheral cystic components exhibiting fluid-debris levels (Figure 2).

**Figure 3:**
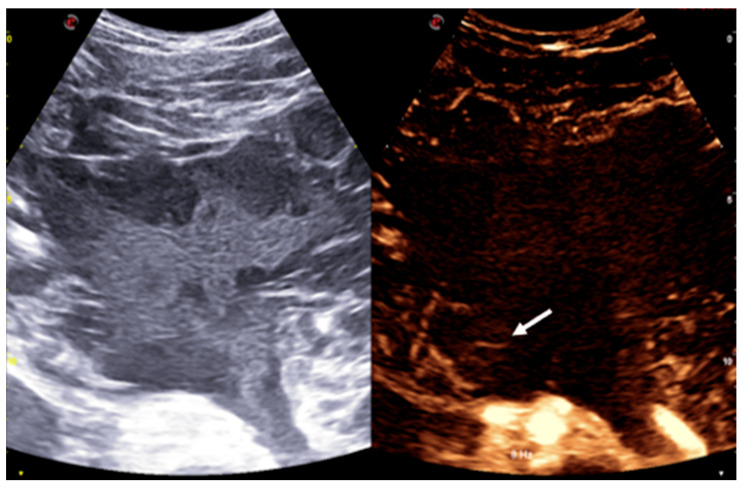
CEUS shows a multicystic lesion composed of individual cysts surrounded by a debris-filled matrix that does not enhance after contrast administration, except for some of the more peripheral septa, which demonstrate mild contrast enhancement (arrow).

Given the imaging characteristics and the broad differential diagnosis, including both benign and malignant entities, an ultrasound-guided biopsy was performed under contrast-enhanced ultrasound (CEUS) guidance. The mass itself showed no contrast uptake, except for some peripheral septa, which exhibited mild contrast enhancement (Figure 3).

Tissue sampling was performed using an 18-gauge needle to obtain material for both histological and cytological evaluation of the previous local anesthesia.

Histological analysis revealed fibroadipose tissue containing smooth muscle elements and blood vessels, without significant cellular atypia. Cytological examination showed amorphous material, occasional pigment-laden macrophages, a moderate number of granulocytes, and a small fragment of fibroadipose tissue with sparse lymphocytic infiltration.

The findings were inconclusive for malignancy or any specific diagnosis.

**Figure 4:**
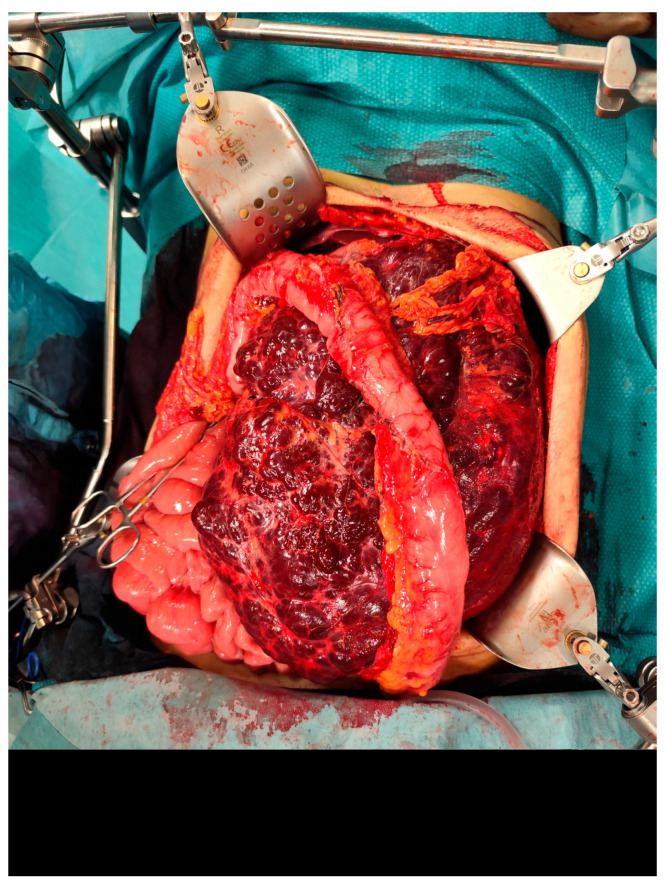
Intraoperative picture showing the mass.

**Figure 5:**
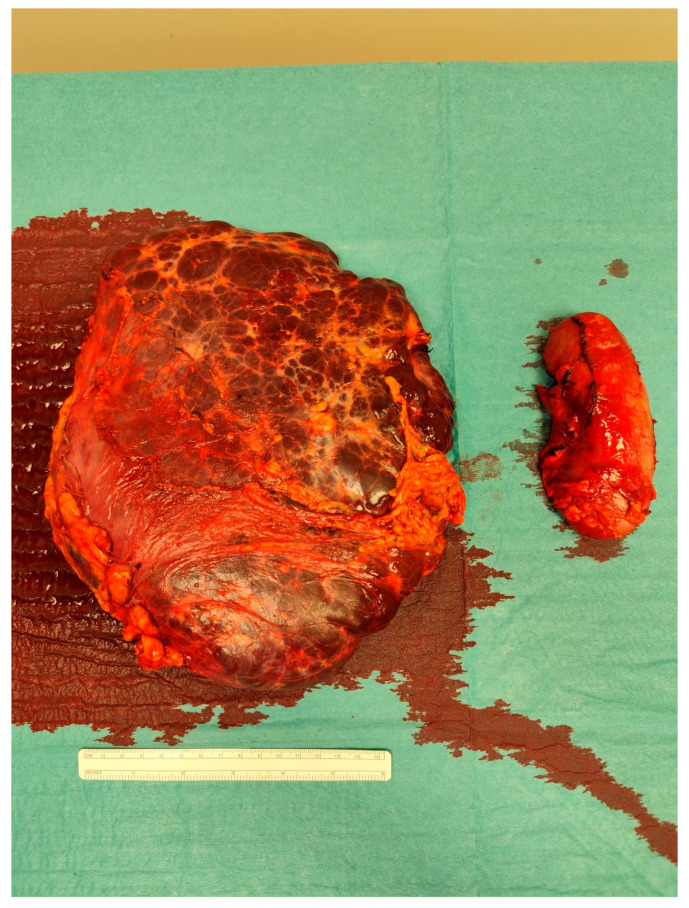
The resected specimen.

The case was discussed during the dedicated multi-disciplinary meeting, and due to the lesion’s considerable size and complex structure, as well as the inconclusive results of the biopsy, surgical resection was advised. The procedure included “en bloc” removal of the left kidney and adrenal gland, both of which were tightly adherent to the mass (Figure 4 and Figure 5).

Postoperative pathological examination revealed a brownish, multiloculated cystic lesion measuring 18 × 17 × 30 cm, with areas of hemorrhage and multiple whitish calcified components. Subsequent analysis identified these calcifications as phleboliths, formed secondary to intralesional thrombotic events.

**Figure 6:**
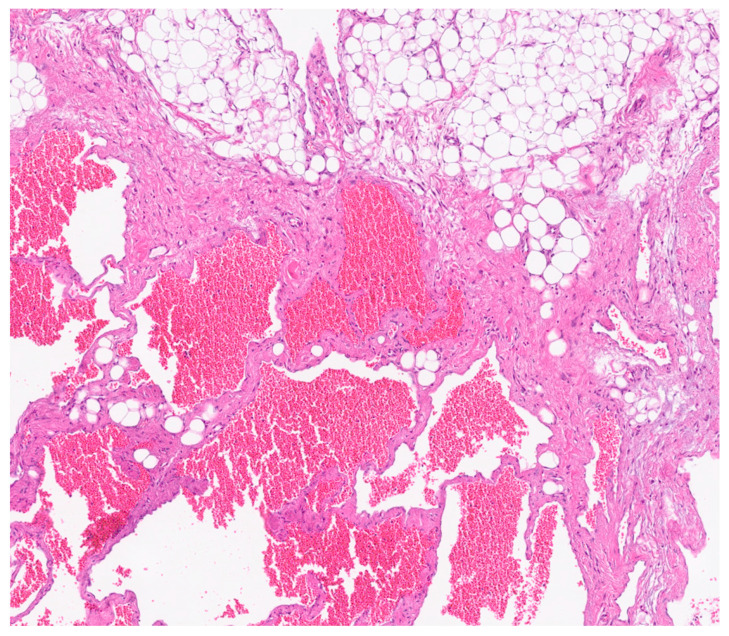
H&E-stained section shows the lesion is composed of variably sized anastomosing vascular spaces.

**Figure 7:**
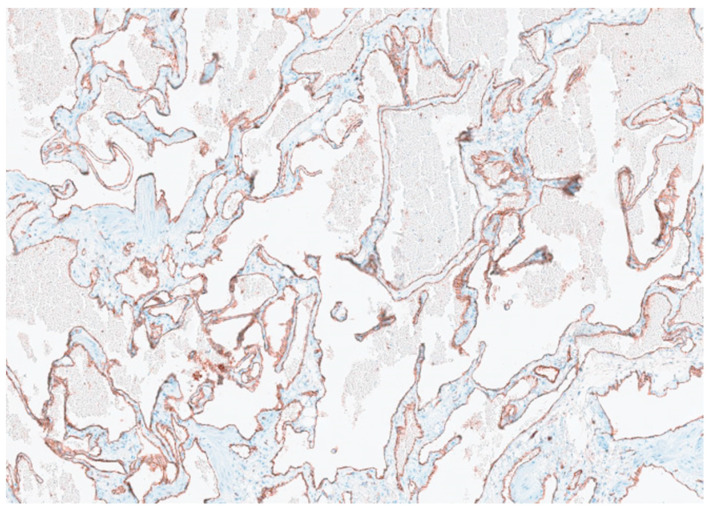
The inner lining cells react to CD31 immunohistochemistry staining.

Immunohistochemical analysis revealed strong positivity for CD31, CD34, and podoplanin (D2-40), highlighting the coexistence of venous and lymphatic endothelial components [5]. This supported the final histological diagnosis of a combined lymphatic-venous malformation, in accordance with the 2018 ISSVA classification [4]. (Figure 6 and Figure 7).

Follow up:

The patient was enrolled in a structured postoperative follow-up program, which included serial abdominal ultrasound examinations performed every three months. Over a total follow-up period of 8 months, all imaging studies demonstrated stable findings, with no evidence of residual or recurrent disease. Clinically, the patient remained asymptomatic throughout the follow-up period, with complete resolution of abdominal distension and associated symptoms. No postoperative complications were reported. At the time of the most recent evaluation, the patient was in good general health. These findings support the effectiveness of complete surgical resection in achieving long-term disease control in cases of large intra-abdominal lymphatic-venous malformations.

## 3. Discussion

The classification system endorsed at the 20th Workshop of the International Society for the Study of Vascular Anomalies (ISSVA) in 2014, and later updated in 2025, differentiates lymphatic malformations into common (cystic) types—macrocystic, microcystic, or mixed—and complex lymphatic anomalies [4].

Lymphatic-venous malformations (LVMs) are uncommon congenital low-flow vascular anomalies characterized by abnormal, interconnected lymphatic and venous vessels [1].

Intra-abdominal lymphatic malformations are rare lymphatic malformations. They account for less than 5% of all lymphatic malformations, with an incidence of 1:250,000 [6].

The most widely accepted hypothesis for their development involves a congenital anomaly. An abnormal connection between lymphatic channels and the venous system during embryogenesis is believed to lead to these vascular anomalies [7].

These malformations can occur anywhere in the body but are most frequently found in regions rich in lymphatic tissue, such as the head and neck, axilla, mediastinum, and groin [1]. Fewer than 5% of LVMs are intra-abdominal [2], typically originating from the retroperitoneum (25%), mesentery (21.9%), retroperitoneum (25%) [8], or greater omentum.

More than 80% of LVMs are diagnosed during the first year of life. They are rarely observed in adult patients. In adulthood, a slight female predominance has been reported, with a sex ratio of approximately 0.6 [8].

The clinical presentation of these anomalies varies widely, depending on the lesion’s size, location, and potential complications. Many patients are asymptomatic, with lesions discovered incidentally during imaging studies or surgeries performed for other reasons. However, others may develop symptoms due to complications such as intracystic bleeding or infection, intestinal volvulus, or mass effect, including ureteric obstruction or hematuria [5].

Given their dynamic nature, LVMs have the potential to enlarge progressively, particularly following episodes of infection or hemorrhage. Their growth may exceed the patient’s overall somatic development and appears to be more pronounced during adolescence, possibly due to hormonal influences, as suggested by the identification of progesterone receptors and the hormone-induced upregulation of VEGF-C within these lesions [9].

The diagnosis of intra-abdominal LVMs can be particularly challenging due to their variable presentation, which can mimic other pathological conditions, including malignant and benign tumors.

Possible malignant causes encompass cystic mesothelioma, teratoma, undifferentiated sarcoma, cystic metastases (notably from ovarian and gastric origins), and malignant mesenchymoma. On the benign side, one might encounter lymphangiomas, cysts stemming from urothelial or intestinal sources, and microcystic pancreatic adenomas. Additionally, several non-cancerous conditions can manifest as cystic lesions, including retroperitoneal hematomas, abscesses, duplication cysts, ovarian cysts, and pancreatic pseudocysts [10]. Imaging is vital for excluding malignancy and understanding the lesion’s anatomical context prior to surgical intervention.

Ultrasound (US) is the preferred initial method for detecting the lesion and determining its size. Following this, computed tomography (CT) and magnetic resonance imaging (MRI) offer more precise images that detail the tumor’s spread, its position, the involvement of nearby organs, and the nature of the fluid within the cystic lesion.

The lesions present distinct imaging characteristics across different modalities. On US, they typically appear as hypoechoic or anechoic multiloculated cystic spaces with thin septa. There is usually no blood flow within the lesion on color Doppler, except in the septa, where high-resistance arterial or venous flow can sometimes be seen [6].

On CT imaging, LVMs typically appear as cystic lesions that may be unilocular or multilocular with internal septations. They usually show fluid attenuation, while the walls and septa often demonstrate contrast enhancement. In some cases, calcifications can be observed, a finding that may also occur in teratomas, as in our case [3].

If infection or hemorrhage occurs, layering echogenic debris on US or increased density on CT may be observed, indicating the presence of blood or other material within the cyst [6].

On MRI, these malformations are usually well-defined, lobulated, and septated. They predominantly show low signal intensity on T1-weighted images and high signal intensity on T2-weighted images. The signal intensity can vary depending on the amount of protein or hemorrhage in the lesion. Occasionally, the content may appear brighter than muscle on T1-weighted images, particularly in cases where the cyst contains protein or blood. Generally, the enhancement after contrast medium agent administration is visible only in the septa and solid components. However, if there is inflammation, the septa surrounding areas may also show significant enhancement [6].

Imaging plays a central role in the diagnosis and management of vascular malformations, providing detailed information on lesion morphology, internal composition, and anatomical relationships, which are essential not only for accurate characterization but also for planning the most appropriate treatment—whether surgical, endovascular, or conservative.

In selected cases, image-guided biopsy may help differentiate benign from malignant lesions. Therefore, a combination of radiologic assessment and histopathological evaluation remains critical for a definitive diagnosis. Immunohistochemistry contributes significantly in this setting by revealing endothelial markers such as CD31, CD34, and podoplanin (D2-40) [5], allowing accurate identification of the lymphatic and venous components according to the ISSVA classification [4].

The management of intra-abdominal LVMs depends on their clinical presentation and morphological classification. Asymptomatic lesions, particularly small-volume macrocystic forms, may be managed conservatively with regular imaging follow-up [11].

Surgery is indicated in symptomatic patients or when there is suspicion of complications such as hemorrhage, infection, or malignancy. Open surgical resection is the preferred approach for intra-abdominal LVMs and remains the most effective treatment to prevent recurrence, provided that complete excision is feasible. However, resectability may be limited by the lesion’s proximity to vital structures.

Sclerotherapy, while successful in treating macrocystic lymphatic malformations in other anatomical locations, has shown variable results in intra-abdominal LVMs and remains controversial due to higher recurrence rates. Nevertheless, it may be considered in selected cases—especially in complex, extensive, or syndromic malformations—either alone or as an adjunct to surgery to reduce lesion volume and improve surgical outcomes.

Accurate classification of LVMs and a multidisciplinary assessment are essential to tailor treatment strategies, balancing efficacy with the potential risks of recurrence or complications [11].

## 4. Conclusions

This case emphasizes the importance of thorough clinical evaluation, including appropriate imaging and histological assessment, in patients presenting with unexplained abdominal distension. Although intra-abdominal lymphatic-venous malformations are extremely rare, they should be considered in the differential diagnosis of large, multiloculated abdominal masses with fluid-like attenuation. While surgical resection is often the preferred and most effective therapeutic approach, it may not always be possible, and in certain circumstances, alternative or supportive treatments should also be taken into account.

## Data Availability

The data are not publicly available due to privacy concerns.
